# Breast cancer incidence and mortality in Georgia by regions and municipalities in 2015-2019: Diana Nemsadze

**DOI:** 10.1093/eurpub/ckac131.012

**Published:** 2022-10-25

**Authors:** V Tkeshelashvili, T Gvazava, T Beruchashvili, E Shvelidze

**Affiliations:** Georgian University, Tbilisi, Georgia

## Abstract

**Background:**

According to the NCDC, in 2015-2019, breast cancer ranked first in the structure of cancer in Georgia. Additional studies are needed to identify breast cancer incidence and mortality by regions and municipalities of Georgia.

**Methods:**

Electronic dBase of cancer population registry for 2015-2019 (52,178 cancer cases), Tbilisi registry database for 2002-2004 (33,478 deaths from all causes), methodology recommended by IARC and UICC, SEER program and 2014 Georgian census data were used in the study. Standardized cancer incidence and mortality rates (ASR, TASR, AAR, SRR, CR64, CR74, SIR, SMR, PIR, 95% CI) were calculated.

**Results:**

29,303 cases of cancer (56% of all cancers) were registered in the Georgian female population in 2015-2019, including 18,432 (62.9%) cases of breast cancer. In the mentioned 5 years, breast cancer ranks first in the general structure of cancer incidence in female population of Georgia and Tbilisi. According to ASR (world standard) and AAR (Georgia and Tbilisi standards, 2014), annual incidence was 62,9%000 /95,7%000 in Georgia, and 85.3%000 /123,6%000 - in Tbilisi. According to the SRR, incidence of breast cancer in Tbilisi was 1.4 times higher than general incidence of breast cancer in Georgia. In 2015-2019, compared to 1988-1992, i.e., in 27-year dynamics, rate of breast cancer in Tbilisi according to the SRR increased 2.4 times, and according to SIR- by 140,2% in dynamics. ASR of breast cancer deaths in Tbilisi in 2015-2019 annually made up 112,2%000. According to the SRR, in 2015-2019, the rate of breast cancer deaths in Tbilisi increased by 3.4 times, compared to 2002-2004. In 13-year dynamics, the incidence of breast cancer deaths according to SMR increased by 238%.

**Conclusions:**

In order to advocate for breast cancer control it is recommended to develop regional and municipal screening programs based epidemiological maps of cancer and ensure effective screening services for each woman through the State program prevention guideline.

**Key messages:**

• Epidemiological maps of breast cancer are recommended for use in planning regional and municipal programs of cancer control, including screening which will help breast cancer control advocacy.

• Ensure effectiveness of screening services for each woman through the State program prevention guideline.

